# New York College of Dentistry

**Published:** 1882-06

**Authors:** 


New York College of Dentistry.—We have received the seventeenth annual announcement of the New York College of Dentistry. This college is the dental department of the University of New York. Among its faculty are Professors F. D. Weisse, Frank Abbott, Alex. W. Stein, F. LeRoy Satterlee, and Dr. Carl Heitzmann. The Trustees state in their circular that "the purpose of the Institution is to educate men to practise dental surgery as a specialty of general surgery; therefore, the fundamental medical sciences applied to the wants of the specialist and the theory of operative and prosthetic dental surgery are lectured upon during the collegiate session. As no amount of scientific attainments can compensate for a lack of skill in practice, the greater part of the student's time is devoted to it."

These principles accord in great measure with the propositions amplified by ourselves in the leading editorial in the January number of this journal, on the *General and Special Education of Dentists.* A close adherence to the course marked out by the faculty of the New York College of Dentistry will unquestionably result in the advancement of the specialty.

